# Clinical outcomes of nephrocalcinosis in preschool-age children: association between nephrocalcinosis improvement and long-term kidney function

**DOI:** 10.3389/fped.2023.1214704

**Published:** 2023-10-09

**Authors:** Hyun Ah Woo, Hyeonju Lee, Young Hun Choi, Jeesu Min, Hee Gyung Kang, Yo Han Ahn, Hyun Kyung Lee

**Affiliations:** ^1^ Department of Pediatrics, Seoul National University Children’s Hospital, Seoul, Republic of Korea; ^2^ Department of Radiology, Seoul National University Hospital, Seoul, Republic of Korea; ^3^ Department of Pediatrics, Chungnam National University Sejong Hospital, Sejong, Republic of Korea; ^4^ Department of Pediatrics, Seoul National University College of Medicine, Seoul, Republic of Korea; ^5^ Kidney Research Institute, Seoul National University Medical Research Center, Seoul, Republic of Korea; ^6^ Department of Pediatrics, Kangwon National University Children's Hospital, Chuncheon, Republic of Korea

**Keywords:** nephrocalcinosis, children, kidney function, tubular disorders, preterm, kidney ultrasonography

## Abstract

**Background:**

We evaluated the long-term clinical outcomes of nephrocalcinosis (NC) according to etiology and grade in preschool-age children with NC.

**Methods:**

We retrospectively analyzed the clinical outcomes and disease grade of children with NC classified into three groups according to etiology: prematurity, tubular disorders, and others.

**Results:**

Overall, 67 children were diagnosed with NC [median age, 0.76 years; interquartile range (IQR) 0.46–2.14 years]. The etiologies of NC included prematurity (28.4%), tubular disorders (25.4%), and others (46.3%). Moreover, 56 (83.6%) children were asymptomatic and diagnosed accidentally through kidney ultrasonography. Newly diagnosed underlying diseases were greater in the tubular disorders group than in the other two groups (*P* = 0.001). Significantly more newly diagnosed NCs were grade 3 than grade 1 (*P* = 0.003). The median estimated glomerular filtration rate (eGFR) changed from 96.1 (IQR 68.8–119.2) ml/min/1.72 m^2^ at diagnosis to 90.9 (IQR 76.4–106.4) ml/min/1.72 m^2^ at the last follow-up, without a significant difference (*P* = 0.096). Changes in the kidney function did not differ according to etiology. However, patients without improvement in NC grade showed a decrease in eGFR from 98.1 (IQR 71.1–132.9) to 87.4 (IQR 74.0–104.1) ml/min/1.73 m^2^ (*P *= 0.023), while patients with improved NC grade did not show any change in the kidney function.

**Conclusions:**

Early recognition, especially in NC grade 3, can help uncover further diagnoses, such as tubular disorders. Long-term kidney function depends on whether the NC grade improves.

## Introduction

Nephrocalcinosis (NC), which is characterized by the deposition of calcium content within the kidney parenchyma or tubular structures, has a multigenic etiology. Various conditions, including hereditary and acquired diseases, can cause increased urinary calcium excretion, which can lead to NC. The etiology of NC differs between adults and children. The most common etiologies of NC in adults include medullary sponge kidney, distal renal tubular acidosis, or primary hyperparathyroidism. In children, however, idiopathic hypercalciuria or hereditary tubulopathies, as well as prematurity, are common causes of NC.

Moreover, children with NC have shown increased incidence of disease progression and stone recurrence. The prognosis of NC largely depends on the underlying causes. In particular, Dent disease, primary hyperoxaluria, or hypomagnesemia with hypercalciuria can progress to kidney failure if not treated effectively (1). As such, it is imperative that the underlying diseases be identified once children are diagnosed with NC.

A few retrospective studies on pediatric NC have been conducted in several centers over the years (2–7). Their results recommended the early diagnosis of underlying diseases for improving kidney outcomes. However, there was insufficient evidence to suggest that the degree of NC was responsible for kidney function or stunted growth, with data suggesting that underlying diseases only mainly affected kidney outcomes. Hence, the current study attempted to identify whether changes in clinical outcomes were present according to NC grade and etiologies. We analyzed the long-term effects of NC grade to better understand the nature of NC in children and its clinical impact on kidney function, growth, and other clinical features.

## Methods

### Study design

We enrolled children who were diagnosed with medullary NC between January 2004 and December 2021 at Seoul National University Children’s Hospital. The inclusion criteria were as follows: children diagnosed under the age of 5 years and having a follow-up period of more than 1 year. All patients were diagnosed with medullary NC based on kidney sonographic findings and classified into the following three groups according to etiology: prematurity group, tubular disorders group, and others group. Prematurity was defined as birth before 37 weeks of gestation age. Tubular disorders group included distal and proximal renal tubular acidosis, Dent diseases, Lowe syndrome, Bartter syndrome, and renal Fanconi syndrome. The others group included those with idiopathic hypercalciuria, Alagille syndrome, neonatal intrahepatic cholestasis caused by citrin deficiency, primary hyperoxaluria, Williams syndrome, congenital hypothyroidism, and immobilization. Only patients who received confirmation through genetic tests such as Sanger sequencing, targeted exome sequencing, or whole exome sequencing were considered as cases of genetic diseases. Patients’ medical records were reviewed to access data on sex, gestational age at birth, onset age, growth, clinical presentation, medication history, kidney sonography, and laboratory findings. These data were obtained, if available, at the time of diagnosis and last follow-up. This study was approved by the Institutional Review Board of Seoul National University Hospital (IRB no. 2208-144-1353).

### Definitions and clinical outcomes

Medullary NC was graded as follows based on kidney sonography: grade 0, no echogenicity; grade 1, mild echogenicity around the medullary pyramid borders; grade 2, moderate echogenicity around and inside the pyramids; grade 3, severe echogenicity of all pyramids (8). Serial NC sonographic grading of individual patients was reviewed by a pediatric radiologist. Glomerular function was estimated as estimated glomerular filtration rate (eGFR), calculated using the Schwartz formula (9). Calciuria was calculated using the spot urine calcium to creatinine (Ca/Cr) ratio (mg/mg). Hypercalciuria was defined per the reference range for each age group. At 0–1 year, urine (Ca/Cr) <0.8 mg/mg of creatinine was considered normal, <0.56 for 1–2 year, <0.5 for 2–3 year, <0.41 for 3–5 year, <0.3 for 5–7 years, <0.25 for 7–10 years, and age 10-17 years <0.24 for 10–17 years of age (10). The height Z score was calculated using the 2017 growth reference for Korean children and adolescents (11). Growth impairment was defined as a height Z score < −1.88. We evaluated long-term clinical outcomes, including changes in eGFR, hypercalciuria, NC grade, and height growth.

### Statistical analysis

Continuous values were expressed as medians with interquartile ranges (IQR). Percentages were expressed as effective percentages in patients with available data. Statistical analyses were performed using the Wilcoxon rank-sum test and the McNemar test for paired samples. Comparisons between the three groups were performed using the Kruskal–Wallis test for continuous variables and Fisher’s exact test for categorical variables. *Post-hoc* analysis to determine which groups differed was conducted using the Mann–Whitney test. For *post-hoc* analysis, Bonferroni’s method was used to determine statistical significance. *P* < 0.05 indicated statistical significance. All data were analyzed using SPSS version 27.0 (IBM, Armonk, NY, USA).

## Results

A total of 67 children (male:female = 40:27) were diagnosed with medullary NC at the median age of 0.76 (IQR 0.46–2.14) years (Table 1), among whom 40 patients were younger than 1 year at the initial diagnosis. The median age at the last follow-up was 7.03 (4.35–11.5) years. The most common cause of NC was a prematurity (*n* = 19). Tubular disorders (*n* = 17) included distal renal tubular acidosis (RTA) (*n* = 7), Lowe syndrome (*n* = 4), Dent disease (*n* = 3), Bartter syndrome (*n* = 2), and renal Fanconi syndrome (*n* = 1). Specific causes of NC are shown in Table 2. Two patients born prematurely who had tubular disorders were included in the tubular disorder category. Additionally, 56 (83.6%) children were asymptomatic and had been diagnosed incidentally through kidney sonography at the median age of 0.73 (IQR 0.44–1.81) years. Only 7 (10.4%) patients had hematuria at the initial diagnosis of NC. In 10 (14.9%) children, the causative diseases leading to NC were newly diagnosed, which included distal RTA (*n* = 3), Dent disease (*n* = 3), Bartter syndrome (*n* = 1), primary hyperoxaluria (*n* = 1), congenital hypothyroidism (*n* = 1), and glycogen storage disease (*n* = 1). Upon diagnosis of NC, 62.7%, 25.4%, and 11.9% of the patients showed grades 1, 2, and 3 on kidney sonography, respectively.

**Table 1:** Baseline characteristics of children with nephrocalcinosis.

	Total (*n* = 67)	Prematurity (*n* = 19)	Tubular disorders (*n* = 17)	Others (*n* = 31)	*P* value
Sex, M:F	40:27	9:10	15:2	16:15	0.021
Age at the diagnosis of NC, years	0.8 (0.5–2.1)	0.4 (0.4–0.7)	1.7 (0.6–3.2)	1.0 (0.6–2.6)	0.003
Follow-up period, years	5.2 (3.2–9.3)	4.2 (2.8–7.4)	8.0 (3.8–12.7)	6.1 (3.1–9.5)	0.225
Initial presentation					0.042
Incidental findings on USG	56 (83.6)	18 (94.7)	11 (64.7)	27 (87.1)	
Urinary symptoms	11 (16.5)	1 (5.3)	6 (35.3)	4 (12.9)	
Newly diagnosed disease	10 (14.9)	0	7 (41.2)	3 (9.7)	0.001
Clinical outcome at the diagnosis of NC					
eGFR, ml/min/1.73 m^2^, *n* = 66	96.1 (68.8–119.2)	95.4 (81.8–107.0)	90.2 (61.1–148.1)	97.4 (67.6–133.1)	0.959
Hypercalciuria, *n* = 66	30 (45.5)	7 (38.9)	10 (58.8)	13 (41.9)	0.440
Height Z score, *n* = 65	−1.38 (−2.50–0.06)	−1.65 (−4.50–0.02)	−1.54 (−2.50 to −0.87)	−1.25 (−2.20–0.02)	0.354
Height Z score < −1.88, *n* = 65	26 (38.8)	9 (47.4)	7 (41.2)	10 (32.3)	0.557
NC grade on USG					0.043
Grade 1	42 (62.7)	16 (84.2)	8 (47.1)	18 (58.1)	
Grade 2	17 (25.4)	3 (15.8)	6 (35.3)	8 (25.8)	
Grade 3	8 (11.9)	0	3 (17.6)	5 (16.1)	
Medication history					
Furosemide	19 (28.4)	7 (36.8)	1 (5.9)	11 (35.5)	0.061
Vitamin D	33 (49.3)	19 (100)	7 (41.2)	7 (22.6)	<0.001
Treatment for nephrocalcinosis					
Thiazide	22 (32.8)	6 (31.6)	7 (41.2)	9 (29)	0.690
Potassium citrate	25 (37.3)	4 (21.1)	9 (52.9)	12 (38.7)	0.143

Values are presented as numbers (effective %) or medians (interquartile ranges).

M, male; F, female; NC, nephrocalcinosis; USG, ultrasonography; eGFR, estimated glomerular filtration rate.

**Table 2:** Underlying conditions of 67 patients with nephrocalcinosis.

	Numbers (%)	Male:female
Prematurity	19 (28.4)	9:10
Extremely preterm (<28 weeks)	13 (19.4)	5:8
Very preterm (28–32 weeks)	5 (7.5)	4:1
Moderate to late preterm (32–37 weeks)	1 (1.5)	0:1
Tubular disorders	17 (25.3)	15:2
Distal renal tubular acidosis	7 (10.4)	5:2
Lowe syndrome	4 (6.0)	4:0
Dent disease	3 (4.5)	3:0
Bartter syndrome	2 (3.0)	2:0
Renal Fanconi syndrome	1 (1.5)	1:0
Others	31 (46.3)	16:15
Idiopathic hypercalciuria	4 (6.0)	2:2
Immobilization	2 (3.0)	1:1
Allagile syndrome	1 (1.5)	1:0
Neonatal intrahepatic cholestasis caused by citrin deficiency	1 (1.5)	0:1
Primary hyperoxaluria	1 (1.5)	1:0
Williams syndrome	1 (1.5)	0:1
Congenital hypothyroidism	1 (1.5)	0:1
4q21 deletion syndrome	1 (1.5)	0:1
Congenital adrenal hyperplasia	1 (1.5)	1:0
Glycogen storage disease	1 (1.5)	1:0
Wilms tumor	1 (1.5)	0:1
Focal segmental glomerulosclerosis	1 (1.5)	0:1
Microvillus inclusion disease	1 (1.5)	0:1
Giant cell hepatitis with autoimmune hemolytic anemia	1 (1.5)	1:0
Opsoclonus-myclonus-ataxia syndrome	1 (1.5)	0:1
Multicystic dysplastic kidney	1 (1.5)	0:1
Sacrococcygeal teratoma	1 (1.5)	0:1
Moyamoya disease	1 (1.5)	1:0
Unknown or no underlying disease	9 (13.4)	7:2

Values are presented as numbers (%).

### Differences in baseline characteristics according to etiology

Sex differences in etiology were observed with the tubular disorders group having significantly more males compared to the other two groups (Table 1, *P* = 0.021). This is because hereditary tubular disorders, such as Dent disease and Lowe syndrome, are X linked. Patients in the prematurity group were diagnosed with NC earlier than those in the other two groups (*P* = 0.003). Although 35.3% of those in the tubular disorders group had urinary symptoms, only a minority of those in the other two groups presented urinary symptoms (*P* = 0.042). The tubular disorders group had significantly more newly diagnosed diseases (41.2%) compared to the other two groups (prematurity group, 0%; others group, 9.7%; *P* = 0.001). NC grade 1 was more prevalent in the prematurity group than in the tubular disorders group (*P *= 0.043). No significant differences in eGFR, hypercalciuria, and height growth were observed between the three groups. No specific differences in furosemide use were noted between the groups (*P* = 0.061). However, vitamin D medication history was significantly higher in the prematurity group than in other two groups (*P* < 0.001).

### Clinical outcomes according to etiology

**Figure 1:**
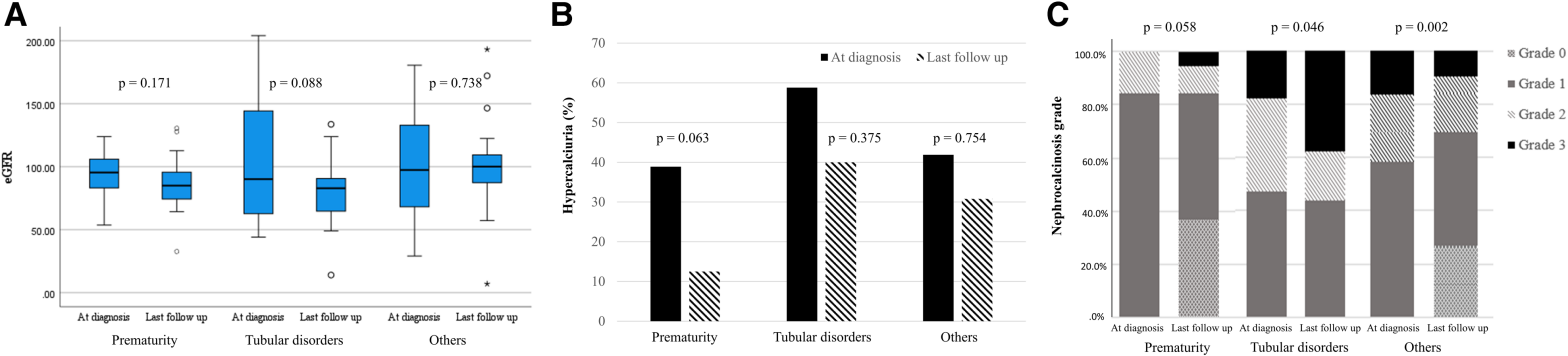
Changes in clinical outcomes according to etiology. (**A**) estimated glomerular filtration rate (ml/min/1.73 m^2^); (**B**) hypercalciuria (%); (**C**) nephrocalcinosis grade.

The median eGFR changed from 96.1 (IQR 68.8–119.2) ml/min/1.73 m^2^ at diagnosis of NC to 90.9 (IQR 76.4–106.4) ml/min/1.73 m^2^ at the last follow-up, without a significant difference (*P* = 0.096). No significant differences in eGFR were observed in all three groups (Figure 1A). To account for potential variations in follow-up duration, we employed a time-normalized change in eGFR to assess the differences between the three groups. The median time-normalized change in eGFR revealed no statistically significant differences among the three groups (*P* = 0.443); −1.086 ml/min/1.73 m^2^ per year (IQR −5.236 to 1.217) in the prematurity group, −3.728 ml/min/1.73 m^2^ per year (IQR −13.350 to 0.465) in the tubular disorders group, and −1.070 ml/min/1.73 m^2^ per year (IQR −9.161 to 4.964) in the others group. At the last follow-up, the tubular disorders group showed significantly worse eGFR the others group (*P* = 0.013). The proportion of hypercalciuria decreased from 45.6% to 28.1% (*P* = 0.041). There were no significant decreases in the proportion of hypercalciuria within each group (Figure 1B). Significant differences in the distribution of NC grade based on kidney ultrasonography were observed between the time of diagnosis and the last follow-up appointment (*P* < 0.001). NC grade changes between the initial and last follow-up were as follows: improved in 27.7%, sustained in 63.1%, and aggravated in 9.2%. Significant differences in NC grade changes were observed according to etiology (*P *= 0.003) (Figure 1C). Notably, the tubular disorder group had worse NC grade outcomes compared to the other two groups (*post-hoc* analysis, tubular disorders group vs. prematurity group, *P *= 0.014; tubular disorders vs. others group, *P* < 0.001). No improvement in NC grade was observed in the tubular disorders group, whereas 36.8% of the prematurity group and 36.7% of the others group showed improvement in NC grade.

Growth impairment defined as a height Z score < −1.88 were found in 26 (38.8%) and 19 (29.2%) patients at diagnosis and the last follow-up, respectively (Supplementary Table S1). No etiological differences in height Z scores and percentage of height Z score < −1.88 were observed between the time of diagnosis and last follow-up appointment (*P *= 0.557 and 0.336, respectively). Half of the 26 children with growth impairment at diagnosis maintained their stunted growth at the last follow-up, whereas only 12 (46.2%) children with initially stunted growth showed catch-up growth. Among the 12 children who successfully achieved catch-up growth, 5 were preterm, 3 had tubular disorders, and 4 had other etiologies. Among the 41 children who did not have growth impairment at diagnosis, 6 developed growth impairment at the last follow-up. Among the six children, one was preterm, three had tubular disorders, and two had other etiologies.

### Kidney outcome according to the nephrocalcinosis grade

**Figure 2:**
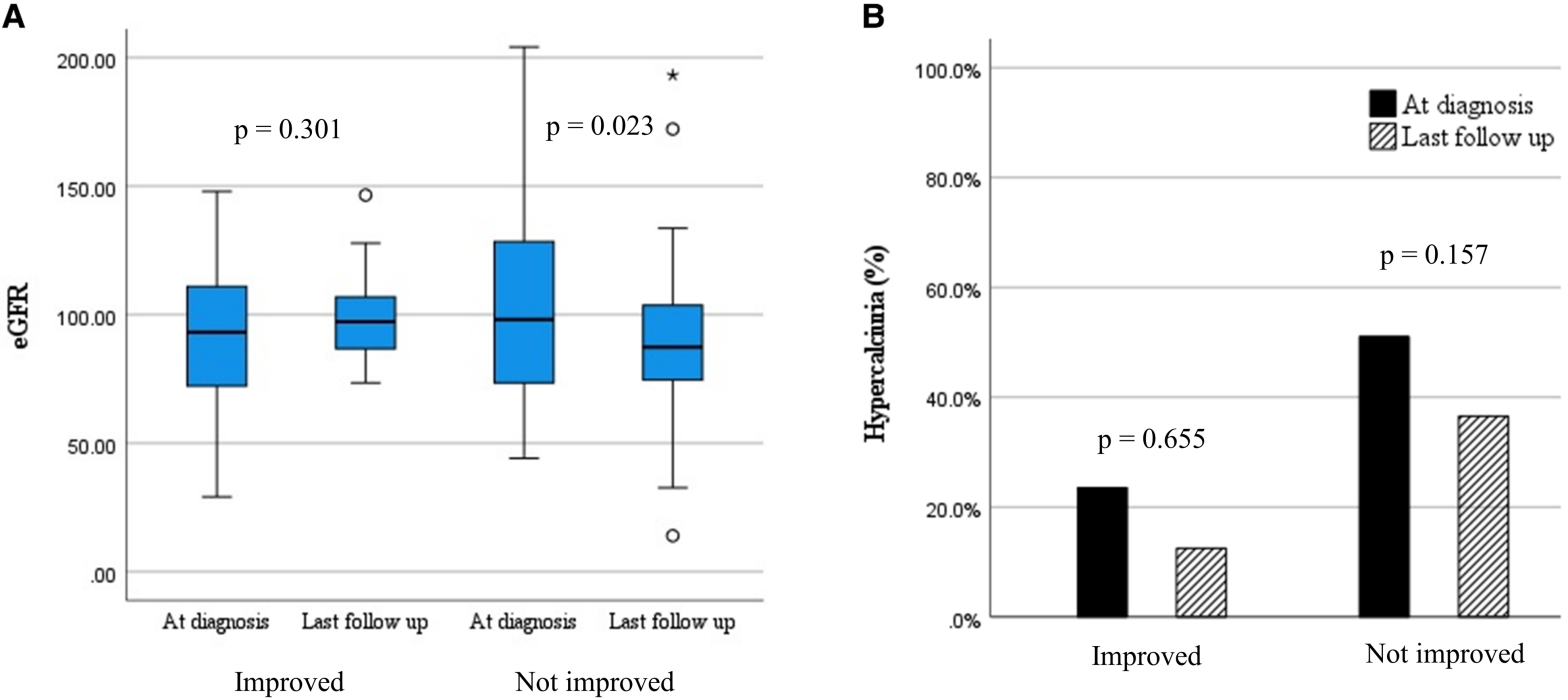
Changes in clinical outcomes according to the change in nephrocalcinosis grade. (**A**) estimated glomerular filtration rate (ml/min/1.73 m^2^); (**B**) hypercalciuria (%).

Significant differences in initial eGFR were observed according to initial NC grade (*P* = 0.008) (Table 3). Post-hoc analysis results showed that those with NC grade 3 had lower eGFR compared to those with NC grade 1 (*P* = 0.002). No significant changes in eGFR were observed according to initial NC grade. However, the median eGFR decreased from 98.1 (IQR 71.14–132.9) to 87.4 (IQR 74.0–104.1) ml/min/1.73 m^2^ (*P *= 0.023) in patients without improvement in NC grade while no change in kidney function was observed in patients with improved NC grade (Figure 2A). Additionally, the median time-normalized change in eGFR revealed a significant difference between patients with or without improved NC grade (1.020 ml/min/1.73 m^2^ per year (IQR −3.120 to 4.960) vs. −2.050 ml/min/1.73 m^2^ per year (IQR −11.570 to 1.280), *P* = 0.031). However, there were no significant changes observed for hypercalciuria in patients with or without improved NC grade (Figure 2B).

**Table 3:** Baseline characteristics and last nephrocalcinosis grade distribution according to initial nephrocalcinosis grade.

	Grade 1 (*n* = 42)	Grade 2 (*n* = 17)	Grade 3 (*n* = 8)	*P* value
Sex, M:F	24:18	12:5	4:4	0.536
Age at the diagnosis of NC, years	0.7 (0.4–2.0)	0.9 (0.7–2.2)	0.9 (0.5–2.5)	0.525
Follow-up period, years	4.3 (3.1–8.2)	7.0 (2.7–10.5)	12.9 (9.5–15.8)	0.004
Initial presentation				0.947
Incidental findings on USG	35 (83.3)	14 (82.3)	7 (87.5)	
Urinary symptoms	7 (16.7)	3 (17.6)	1 (12.5)	
Newly diagnosed disease	2 (4.8)	4 (23.5)	4 (50.0)	0.003
Clinical presentation at the diagnosis of NC				
eGFR, ml/min/1.73 m^2^, *n* = 66	98.1 (82.3–128.4)	97.9 (62.9–112.4)	56.3 (45.6–70.4)	0.008
Hypercalciuria, *n* = 66	18 (43.9)	8 (47.1)	4 (50)	0.880
Height Z score, *n* = 65	−1.15 (−2.34–0.03)	−1.55 (−2.63 to −1.14)	−1.67 (−2.36–0.07)	0.473
Height Z score < −1.88, *n* = 65	16 (38.1)	7 (41.2)	3 (37.5)	0.973
NC grade on USG at the last follow-up, *n* = 65				<0.001
Grade 0	14 (33.3)	1 (6.7)	0	
Grade 1	26 (61.9)	2 (13.3)	1 (12.5)	
Grade 2	2 (4.8)	8 (53.3)	1 (12.5)	
Grade 3	0	4 (26.7)	6 (75)	
Medication history				
Furosemide	13 (31.0)	4 (23.5)	2 (25)	0.830
Vitamin D	26 (61.9)	5 (29.4)	2 (25)	0.028
Treatment for nephrocalcinosis				
Thiazide	14 (33.3)	5 (29.4)	3 (37.5)	0.918
Potassium citrate	12 (28.6)	8 (47.1)	5 (63.5)	0.124

Values are presented as numbers (effective %) or medians (interquartile ranges).

M, male; F, female; NC, nephrocalcinosis; USG, ultrasonography; eGFR, estimated glomerular filtration rate.

## Discussion

To the best of our knowledge, this has been the first study on preschool-age children with NC who had been followed for a long period of time, allowing us to thoroughly determine long-term effects of NC on various clinical aspects. Most patients with NC were asymptomatic, and in some cases, the underlying disorders were newly diagnosed, especially tubular disorders. A significant decrease in kidney function occurred in the absence of NC grade improvement. These findings suggest the necessity of monitoring long-term kidney function and NC grade in preschool-age children with NC.

NC is mostly asymptomatic and often diagnosed when sonography is performed for other reasons, especially during early childhood. In the current study, 56 (83.6%) patients were diagnosed with NC incidentally on sonography without any urinary symptoms. The proportion of hypercalciuria, a known risk factor for NC, was similar between patients with incidentally found NC (47.3%) and the whole study population (45.5%). A seventh of the children were newly diagnosed with underlying disorders after being diagnosed with NC. Among them, 80% had genetic kidney disorders, such as distal RTA, Dent disease, Batter syndrome, and primary oxaluria. Considerable evidence has shown that children with NC have several underlying genetic disorders (12). However, no specific genetic tests are available to determine when children have NC. Hence, clinicians should consider the possibility of an underlying disease in children with NC incidentally discovered on sonography.

Preterm infants could develop NC due to an immature kidney and various injuries after birth (13, 14). The current study showed that no significant change in eGFR was observed among preterm infants with nephrocalcinosis. However, it remains uncertain whether NC is associated with decreased kidney function in preterm infants. A prospective observational study revealed that up to 60% of preterm infants with NC had long-term sequelae for glomerular and tubular function with low plasma bicarbonate and high urine calcium/citrate ratios (15). In contrast, two case–control studies showed no differences in long-term kidney outcomes between preterm infants with NC and without NC (16, 17). Fayard et al. reported that NC resolved in 61% of preterm infants within 12–18 months after diagnosis (13).

NC is common in patients with kidney tubular disorders that cause hypercalciuria, tubular injury, or crystal nucleation (18, 19). The tubular disorders group had the worst kidney function outcomes and NC grade among the studies groups at last the follow-up and showed sustained or aggravated NC grade, a finding consisted with the results of previous retrospective survey (2). Previous studies have shown that kidney function depended on the underlying disease in children with NC (2, 3, 6, 7). NC is a risk factor for chronic kidney disease (CKD) progression in kidney tubular disorders, especially Lowe syndrome and Dent disease (20). Given that most patients with Dent disease develop kidney failure in their forties, determining the relationship between NC and eGFR is imperative (20). Evidence has shown that treatment of underlying distal RTA prevents progression of NC and deterioration of kidney function (21). Hence, early diagnosis and appropriate management are needed to slow the progression of CKD in children with NC caused by tubular disorders.

The current study analyzed clinical outcomes according to initial NC grade and changes therein. Notably, no differences in kidney function changes were observed according to the initial NC grade at diagnosis. Although patients with improved NC grade maintained their kidney function, those with sustained or aggravated NC grade showed a significant decrease in kidney function. Our findings showed that the change in NC grade rather than the initial NC grade affected long-term kidney function. To date, there have been few reports evaluating changes in kidney function according to changes in NC grade. Among such studies, Ronnefarth et al. showed that children with NC grade 3 presented a significantly lower GFR and urinary osmolality (2). Most patients (72.3%) with NC included herein showed no improvement in NC grade. None of the patients with initial grade 3 NC achieved disease clearance, with 75% maintaining their original NC grade 3. These findings suggest that early diagnosis and treatment of NC before it becomes severe is important in preventing the severity of NC and deterioration of kidney function in children with NC. However, the underlying disease, especially tubular disorders, has the potential to contribute to CKD. The highest persistence in NC grade was observed in the tubular disorders group. Therefore, it remains unclear whether this effect is directly related to NC or attributed to the underlying disease.

## Conclusion

Our analysis of available data allowed us to determine the long-term outcomes of NC in preschool-age children. This study focused on differences in clinical manifestation according to etiology and change in NC grade kidney ultrasonography. Overall, our findings suggest the need for careful monitoring of kidney function especially in those who show no improvement in NC grade given the decrease in long-term kidney function observed in such populations.

## Data Availability

The original contributions presented in the study are included in the article/**Supplementary Material**, further inquiries can be directed to the corresponding authors.
