# Supplementary material for: Clinical outcomes of nephrocalcinosis in preschool-age children: association between nephrocalcinosis improvement and long-term kidney function
Source: Front Pediatr. 2023 Oct 9;11:1214704. doi: 10.3389/fped.2023.1214704 (PMC10603223; doi:10.3389/fped.2023.1214704)
Supplement: Supplementary file 1 [file Table1.docx]

**A Supplementary Table**

Title: Clinical outcomes of nephrocalcinosis in preschool-age children

: association between nephrocalcinosis improvement and long-term kidney function

Frontiers in Pediatrics - Pediatric Nephrology

**Authors**

Hyun Ah Woo, Hyeonju Lee, Young Hun Choi, Jeesu Min, Hee Gyung Kang, Yo Han Ahn, Hyun Kyung Lee

**Corresponding Authors**

Yo Han Ahn, MD, PhD

Department of Pediatrics

Seoul National University Children’s Hospital

101 Daehak-ro, Jongno-gu, Seoul 03080, Republic of Korea

Tel.: +82-2-2072-3350; Fax: +82-2-2072-0633; E-mail: yhahn@snu.ac.kr

Hyun Kyung Lee

Department of Pediatrics

Kangwon National University Children’s Hospital

1 Kangwondaehak-gil, Chuncheon-si, Gangwon-do, Republic of Korea

Tel.:+82-33-258-4969; Fax:+82-33-258-9329; E-mail: hkped@naver.com

Supplementary Table 1. Changes of height z-score according to etiology and initial NC grade

|  | At diagnosis | Last follow-up | P value |
| --- | --- | --- | --- |
| ***Total (n=67)*** |  |  |  |
| Height Z score (n=65) | -1.38 (-2.50 – 0.055) | -1.18 (-2.40 – -0.10) | 0.488 |
| Height Z score <-1.88 (n=65) | 26 (38.8) | 19 (29.2) | 0.157 |
| Etiological classification | | | |
| ***Preterm (n=19)*** |  |  |  |
| Height Z score (n=19) | -1.65 (-4.5–0.02) | -1.46 (-2.3–-0.93) | 0.601 |
| Height Z score <-1.88 (n=19) | 9 (47.4) | 5 (26.3) | 0.102 |
| ***Tubular disorders (n=17)*** |  |  |  |
| Height Z score (n=16) | -1.54 (-2.5–-0.87) | -1.43 (-3.51–-0.41) | 0.918 |
| Height Z score <-1.88 (n=16) | 7 (41.2) | 7 (43.8) | 1.000 |
| ***Others (n=31)*** |  |  |  |
| Height Z score (n=30) | -1.25 (-2.2–0.02) | -1.43 (-3.51–-0.41) | 0.344 |
| Height Z score <-1.88 (n=30) | 10 (32.3) | 7 (23.3) | 0.414 |
| Initial NC grade classification | | | |
| ***Grade 1 (n=42)*** |  |  |  |
| Height Z score (n=41) | -1.15 (-2.34–0.03) | -1.14 (-2.76– -0.45) | 0.836 |
| Height Z score <-1.88 (n=41) | 16 (38.1) | 14 (34.1) | 0.763 |
| ***Grade 2 (n=17)*** |  |  |  |
| Height Z score (n=16) | -1.55 (-2.63– -1.14) | -1.44 (-2.00 - -0.61) | 0.352 |
| Height Z score <-1.88 (n=16) | 7 (41.2) | 4 (23.5) | 0.18 |
| ***Grade 3 (n=8)*** |  |  |  |
| Height Z score (n=8) | -1.67 (-2.36–0.07) | -0.5 (-1.39–0.55) | 0.123 |
| Height Z score <-1.88 (n=8) | 3 (37.5) | 1 (12.5) | 0.157 |

Values are presented as number (effective %) or median (interquartile ranges).

NC, Nephrocalcinosis; IQR, interquartile ranges
